# Effectiveness of Telerehabilitation on Motor Impairments, Non-motor Symptoms and Compliance in Patients With Parkinson's Disease: A Systematic Review

**DOI:** 10.3389/fneur.2021.627999

**Published:** 2021-08-26

**Authors:** Chiara Vellata, Stefano Belli, Francesca Balsamo, Andrea Giordano, Roberto Colombo, Giorgio Maggioni

**Affiliations:** ^1^Istituti Clinici Scientifici Maugeri Spa – Società Benefit, Neurologic Rehabilitation Unit of Veruno Institute, Veruno, Italy; ^2^Istituti Clinici Scientifici Maugeri Spa – Società Benefit, Bioengineering Service, Veruno, Italy

**Keywords:** Parkinson's disease, review, remote physical activity, digital health, telerehabilitation, online rehabilitation

## Abstract

**Introduction:** Parkinson's disease (PD) is a chronic neurodegenerative disease involving a progressive alteration of the motor and non-motor function. PD influences the patient's daily living and reduces participation and quality of life in all phases of the disease. Early physical exercise can mitigate the effects of symptoms but access to specialist care is difficult. With current technological progress, telemedicine, and telerehabilitation is now a viable option for managing patients, although few studies have investigated the use of telerehabilitation in PD. In this systematic review, was investigated whether telerehabilitation leads to improvements in global or specific motor tasks (gait and balance, hand function) and non-motor dysfunction (motor speech disorder, dysphagia). The impact of TR on quality of life and patient satisfaction, were also assessed. The usage of telerehabilitation technologies in the management of cognitive impairment was not addressed.

**Method:** An electronic database search was performed using the following databases: PubMed/MEDLINE, COCHRANE Library, PEDro, and SCOPUS for data published between January 2005 and December 2019 on the effects of telerehabilitation systems in managing motor and non-motor symptoms. This systematic review was conducted in accordance with the PRISMA guideline and was registered in the PROSPERO database (CRD42020141300).

**Results:** A total of 15 articles involving 421 patients affected by PD were analyzed. The articles were divided into two categories based on their topic of interest or outcome. The first category consisted of the effects of telerehabilitation on gait and balance (3), dexterity of the upper limbs (3), and bradykinesia (0); the second category regarded non-motor symptoms such as speech disorders (8) and dysphagia (0). Quality of life (7) and patient satisfaction (8) following telerehabilitation programs were also analyzed, as well as feasibility and costs.

**Conclusion:** Telerehabilitation is feasible in people affected by PD. Our analysis of the available data highlighted that telerehabilitation systems are effective in maintaining and/or improving some clinical and non-clinical aspects of PD (balance and gait, speech and voice, quality of life, patient satisfaction).

**Systematic Review Registration:**https://www.crd.york.ac.uk/prospero/, identifier: CRD42020141300.

## Introduction

Parkinson's disease (PD) is the second most common neurodegenerative disorder (1) and the most common movement disorder worldwide (1, 2). The prevalence of PD increases steadily with age: in industrialized countries, it affects 0.3% of the entire population, about 1% of people over 60 years of age and 3% of those older than 80 years (1–4); annual incidence rates of PD are estimated between 8 and 18 per 100,000 (4). Statistically, the prevalence varies according to different factors. First, geographical location—PD prevalence is significantly lower in Asia than in North America, Europe, and Australia (1, 5). In Europe, estimated prevalence ranges between 65 and 12,500 per 100,000 inhabitants, while annual incidence rates are estimated between 5 and 346 per 100,000 (6). Another factor affecting PD prevalence is sex—males are more affected than females (7).

PD is a chronic neurodegenerative disease of the extrapyramidal system that affects the central nervous system and involves a progressive alteration motor and non-motor function (8). Pathologically, PD is a consequence of the depletion of dopaminergic neurons in the *pars compacta* of the *substantia nigra*, components of the basal ganglia (2). The key motor symptoms go by the acronym TRAP (9): (1) Tremor, which is the primary disorder in 70% of PD patients; (2) Rigidity (or stiffness); (3) Akinesia/bradykinesia; and (4) Postural control/postural instability. TRAP associated with flexed posture and freezing are the main motor dysfunctions in patients affected by PD (2). Although PD is typically a motor disorder, it is also characterized by non-motor symptoms (2, 10, 11). Non-motor dysfunctions include a wide range of symptoms: speech and communication disorders (dysarthria) (12); autonomic dysfunction (13) of the gastrointestinal (dysphagia, sialorrhea) (14), urinary (15), and cardiovascular (16) systems; sleep problems (17); sensory features: olfactory and visual deficits, pain and somatosensory disturbances (11, 18); neuropsychiatric symptoms: anxiety (19), apathy and fatigue (20), depression (21) and, finally, cognitive impairments, and dementia (22). Together, motor and non-motor symptoms play a decisive role in patients' disability and worsen their quality of life (QoL).

PD is characterized by a relatively slow progression. The therapies available to date offer a good response in the control of motor symptoms, but lose their effectiveness during the natural course of the disease, particularly in the advanced stages when non-motor symptoms become more evident (10, 23, 24). Alongside drug therapy, research shows that early physical exercise is beneficial for PD patients and an early start of rehabilitation is highly recommended, even in the initial stages of the disease (25–27). Regular physical exercise and appropriate training in a multidisciplinary setting can significantly improve motor function, postural control, balance, and strength in PD patients (28), improving clinical outcomes (29). Treatment can mitigate the effects of symptoms, reduce the progression of PD, and prolong the patient's autonomy. The success of PD treatment depends not only on the quality of treatment, but also on the timing and frequency of interventions (30). Intzandt et al. (31) analyzed how different types of training—aerobic, resistance, and goal-based—can influence motor function (gait) and cognition. Their review highlighted the potential for exercise-driven mechanism to improve gait and cognition. Research has shown that appropriate physical exercise can mitigate some of the non-motor symptoms of PD such as fatigue, depression, apathy, and cognitive impairment, all symptoms that can also influence negatively the motor performance (24).

However, access to PD specialists is difficult, with transportation barriers resulting in additional costs for those who do not live near a specialty clinic, and creating a potential health risk for patients (29). In recent years, the use of technologies in various clinical settings has progressed considerably and, with the development of telemedicine systems, telehealth has now become a viable option for managing patients with PD (32–34). Telecommunications and virtual technologies are the tools with which telemedicine functions to provide health support outside traditional health settings. At the base of digital health, which uses information and communication technologies (ICTs), there is telemedicine. Well-designed telehealth schemes can improve health care access and outcomes, especially for chronic disease and fragile groups. Telemedicine refers to the remote delivery of health care services (by all health care professionals) where distance is a critical factor. As the World Health Organization (WHO) states, “telemedicine should include diagnosis, monitoring, treatment and prevention of disease and injuries, research and evaluation, as well as the continuing education of health care providers, all in the interests of advancing the health of individuals and their communities.” The ability to provide technological health services has been made possible by the increase in digital availability and almost free internet access. Telemedicine offers an innovative approach to increase access to clinical rehabilitation medicine services, particularly for people with geographic or mobility limitations (35). Through remote rehabilitation systems, called Telerehabilitation (TR) (36), services can be provided to users at reduced cost and time. TR aims to improve the QoL and daily life autonomy of patients (35–38). Galea (39) showed how TR can strengthen the patient-provider connection by (i) enhancing the health care providers' knowledge about the patients and their contextual factors; (ii) providing information exchange and facilitating patient education; and (iii) establishing shared goal setting and action planning. In the inpatient setting, TR has been used to shorten the hospital stay, facilitate discharge home, and provide patient and caregiver education and support.

The patient approach with TR has proven to be as effective as face-to-face treatment in different clinical conditions such as chronic cardiac disease, neurological dysfunction, and musculoskeletal disorders (40). In addition, TR may not just be comparable to but it may be more effective than traditional rehabilitation, in that it provides new opportunities for increasing accessibility and creating a less restrictive environment (37).

Telemedicine and TR are particularly suitable for patients with PD. Recent clinical trials and some meta-analyses indicate that specific treatments, mostly based on VR systems delivered by TR, are feasible and can offer clinical benefits and outcomes that are comparable to inpatient care, with potential time- and cost-savings (33, 38). In recent years, virtual reality (VR) systems with exercise-based computer activities and video monitoring have been introduced in the management of PD. VR applications allow the user to enter a simulated environment through multimodal sensory feedback. VR-based programs (with or without TR) have led to improvements in sensory strategies (i.e., sensorimotor integration and reweighting) and improved the ability to integrate and reweight the incoming sensory inputs and shape the system of coordinates on which the body's postural control is based (41). Finally, VR-based exercise programs can elicit the integration of motor and cognitive abilities (i.e., attention, executive functions) and stimulate the brain's reward circuitry. VR engages participants in cognitive and motor activities (i.e., dual tasking) that require planning, attention, sensory integration, and processing of stimuli from the virtual environment (42). Recently, videoconferencing technologies have become more available, more precise, and less expensive. Continuation of exercise therapy with TR programs at home could be an acceptable solution in cases where intensified exercising and prolonged periods of training are required. TR can be used both as an alternative to traditional inpatient, outpatient, or home care and as an integration to these care modes (35). Use of a specific TR protocol enables a larger group of patients to perform a task, at the same time and with less healthcare personnel than in clinical settings. Studies have been carried out using TR as a treatment strategy for different clinical disorders, e.g., neurologic disease [stroke (43, 44), spinal cord injury (45), multiple sclerosis (46, 47)], cardiopulmonary disease (coronary artery, congestive heart failure) (48), musculoskeletal dysfunctions (49, 50) and chronic pain and rheumatic diseases (51). For example, Finkelstein et al. (52) in their pilot study in 12 patients with multiple sclerosis report that TR resulted in improvements in gait and balance at the 25-foot walk, 6-min Walking Test (6mWT), and Berg Balance Scale (BBS). Another study by Marshall et al. (53) showed TR to be effective in individuals with lung problems who did not have access to treatment.

Although telemedicine (and TR) is now widely accepted as an appropriate model for delivery of health professional services in the field of physical therapy, with already established standards, guidelines, and policies, there are still few studies in the literature on the use of TR as a rehabilitation tool in people affected by PD. In this systematic review, the use of TR as a treatment approach for motor and non-motor symptoms in people affected by PD were investigated, specifically gait and balance, dexterity of the upper limbs, bradykinesia, and dysphagia and speech disorders, respectively. The usage of TR technologies in the management of cognitive impairment was not addressed. Although these deficits are involved in non-motor disorders related to PD, we consider this topic too wide to cover in a single study—it deserves a dedicated work.

The aim of this systematic review was to investigate whether TR leads to improvements in global or specific motor tasks (gait and balance, hand function) and non-motor dysfunction (motor speech disorders, dysphagia). The impact of TR on QoL and patient satisfaction, as well as its feasibility and costs, were also assessed.

## Methods

This systematic review was conducted according to the Preferred Reporting Items for Systematic Reviews and Meta-Analyses (PRISMA) guidelines and flow diagram (54). The protocol is registered in the International Prospective Register of Systematic Reviews (PROSPERO) under the registration number CRD42020141300.

### Data Sources

An electronic database search was performed from 1st September to 31st December 2019 using the following databases: PubMed/MEDLINE, COCHRANE Library, PEDro, and SCOPUS. In line with the PRISMA guidelines, we then performed an additional manual search (e.g., through citations of articles included in this review). All databases were searched from the establishment of the database (January 2005) to 31 December 2019. The search strategy used a combination of MeSH (Medical Subject Headings) terms and keywords including “Parkinson's disease,” “Telerehabilitation,” “TR,” “Remote Rehabilitation,” “Home-based Rehabilitation,” and their related synonyms. The detail strategy is presented in Appendix 1. The PICOS principle (Population, Intervention, Comparison, Outcome measures, and Study design) was followed to define our research main question—Table 1.

**Table 1:** PICOS design.

**PICOS**	
Population	Parkinson
Intervention/indicator	Telerehabilitation; Remote rehabilitation; Home-based rehabilitation
Comparator/Control	Not using the device, face-to-face
Outcome	Effectiveness of telerehabilitation; Patient satisfaction
Study	Review

### Study Selection Process

Two authors (C.V., S.B.) conducted the literature search independently. First, they manually identified and excluded duplicate references, and then screened titles and abstracts for relevance. The eligibility criteria were agreed by consensus when comparing search results (C.V., S.B.). They were (*i*) the use of remote monitoring via computer devices and wearable sensors to treat PD patients at home, (*ii*) recording of human physical activity, (*iii*) adults aged 18–80 years with PD diagnosis, (*iv*) data published between January 2005 and December 2019, and (*v*) randomized controlled trial (RCT), review and systematic review, case study or series—Table 2. The exclusion criteria were (*i*) no use of wearable/portable sensors/TR system, (*ii*) management of other types of neurological dysfunction or musculoskeletal disorders, (*iii*) focus on cognitive deficit management with TR, (*iv*) article type = abstract, letter, poster or chapter from a book, (*v*) article not written in English, and (*vi*) full access to article not available—Table 3.

**Table 2:** Inclusion criteria.

**Inclusion criteria**
1) Use of remote monitoring via computer devices and wearable sensors to treat PD patients at home
2) Recording of human physical activity
3) Adults aged 18–80 years with PD diagnosis
4) Data released between January 2005 and December 2019
5) Randomized Controlled Trial (RCT), review and systematic review, case study/series

**Table 3:** Exclusion criteria.

**Exclusion criteria**
1) No use of any type of wearable/portable sensors/TR system
2) Management of other types of neurological dysfunction or musculoskeletal disorders
3) Focus on cognitive deficit management with TR
4) Article type (abstract, letter, poster or chapter from a book)
5) Article not written in English
6) Full access not available to article

### Data Extraction and Analysis

Data were extracted (C.V.) and checked (S.B., F.B., G.M.) with final adjudication by consensus. Variables extracted included the population sample studied, disease-specific severity levels, TR device name, intervention, setting, demographic data and study details, including design, funding sources, and motivational factors.

## Results

### Analysis Methods

**Figure 1:**
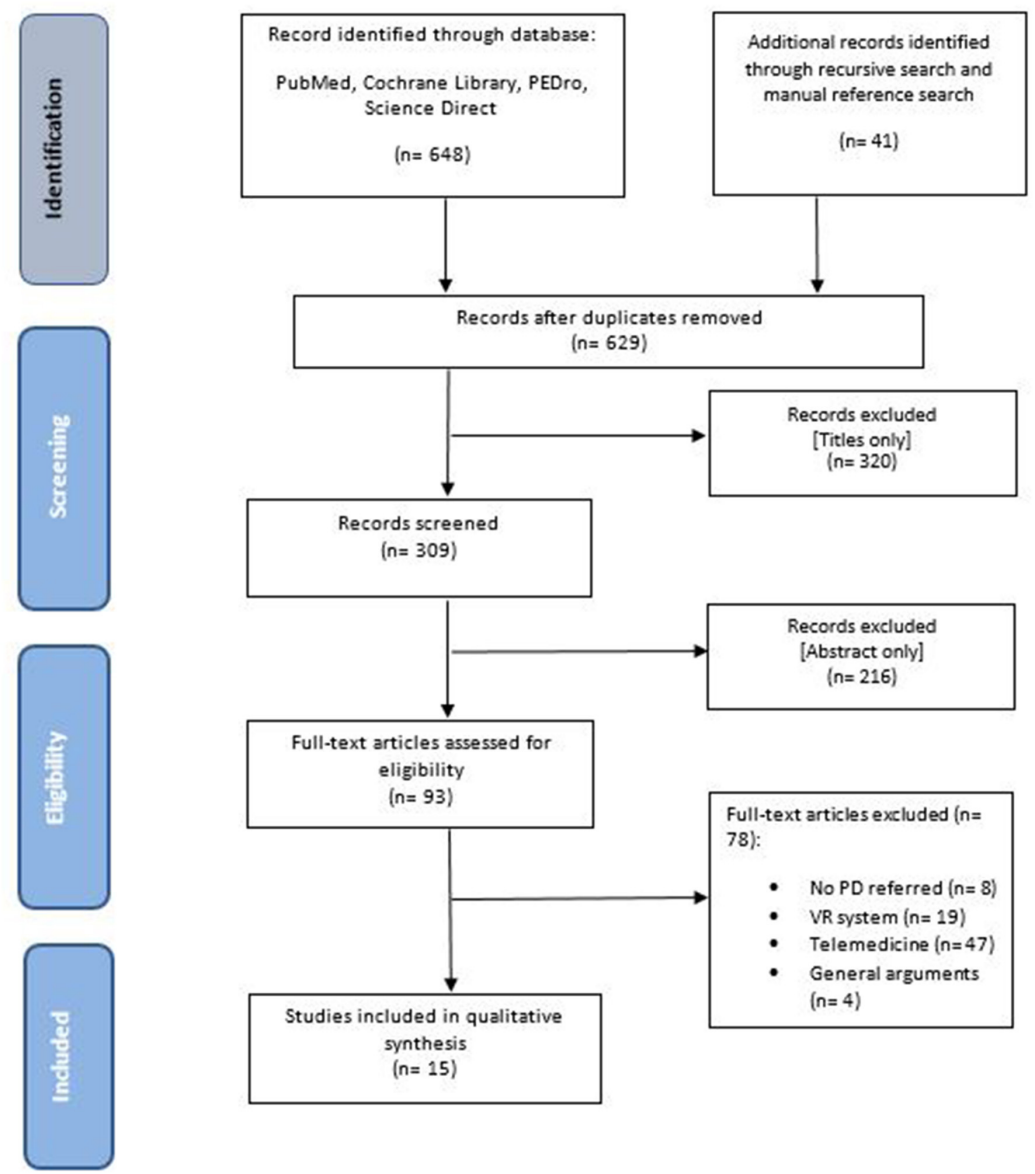
Flow chart.

The search identified 689 articles: 258 were retrieved from PubMed Central, 85 from COCHRANE Library, 1 from PEDro, 304 from Science Direct, while 41 papers were identified by an additional manual search. After removing duplicate items (60), 629 articles remained. Of these, 320 were excluded based on the title only. Of the remaining 309 articles, a further 216 records were excluded after reading the abstract. We fully assessed the full texts of the remaining 93 papers according to the eligibility criteria, and 15 articles met the study criteria and were included in the final analysis (Figure 1). These 15 articles, involving 421 patients affected by PD, consisted of 8 RCTs (42, 55–61), 1 clinical trial (62), 2 case studies (63, 64), 3 pilot studies (65–67), and 1 study design (68).

### Overview of TR Use

The selected articles were divided into two categories based on their topic of interest or outcome.

The first category dealt with the effect of TR on gait and balance (3), dexterity of the upper limbs (3), and bradykinesia (0). The second category dealt with the effect of TR on non-motor symptoms such as speech disorders (8) and dysphagia (0). We also analyzed QoL (7) and patient satisfaction (8) with the TR program, as well as feasibility and costs—Table 4.

**Table 4:** Focus on points of interest of the included articles.

**Title**	**Author**	**Study**	**Focus**								
			**GB**	**ULd**	**Bk**	**SV**	**Dy**	**QoL**	**Sat**	**Feas**	**Cost**
Virtual Reality Telerehabilitation for Postural Instability in Parkinson's Disease: A Multicenter, Single-Blind, Randomized, Controlled Trial	Gandolfi et al. (42)	RCT	✓					✓	✓		✓
Feasibility and preliminary efficacy of a telerehabilitation approach to group adapted tango instruction for people with Parkinson disease	Seidler et al. (55)	RCT	✓							✓	
Use of a Telehealth System to Enhance a Home Exercise Program for a Person With Parkinson Disease: A Case Report	Chatto et al. (63)	Case study	✓					✓	✓	✓	
Can telerehabilitation games lead to functional improvement of upper extremities in individuals with Parkinson's disease?	Cikajlo et al. (56)	RCT		✓				✓		✓	
Using the Internet to assess activities of daily living and hand function in people with Parkinson's disease.	Hoffmann et al. (57)	RCT		✓							
An Internet-Based Telerehabilitation System for the Assessment of Motor Speech Disorders: A Pilot Study	Hill et al. (65)	Pilot Study				✓				✓	
Delivering the Lee Silverman Voice Treatment (LSVT) by web camera: a feasibility study	Howell et al. (66)	Pilot Study				✓				✓	
Home-based speech treatment for Parkinson's disease delivered remotely: a case report	Constantinescu et al. (64)	Case Study				✓			✓	✓	
Assessing disordered speech and voice in Parkinson's disease: a telerehabilitation application	Constantinescu et al. (58)	RCT				✓			✓	✓	
Treating disordered speech and voice in Parkinson's disease online: a randomized controlled non-inferiority trial	Constantinescu et al. (59)	RCT				✓			✓		
Clinical and Quality of Life Outcomes of Speech Treatment for Parkinson's Disease Delivered to the Home Via Telerehabilitation: A Non-inferiority Randomized Controlled Trial	Theodoros et al. (60)	RCT				✓		✓			
The effectiveness of Lee Silverman Voice Treatment therapy issued interactively through an iPad device: a non-inferiority study	Griffin et al. (62)	Trial				✓			✓	✓	✓
Delivering group speech maintenance therapy via telerehabilitation to people with Parkinson's disease: A pilot study	Quinn et al. (67)	Pilot study				✓		✓	✓		
ParkProTrain: an individualized, tabletbased physiotherapy training programme aimed at improving quality of life and participation restrictions in PD patients—a study protocol for a quasi-randomized, longitudinal and sequential multi-method study	Siegert et al. (68)	Study design						✓			
High patient satisfaction with telehealth in Parkinson disease A randomized controlled study.	Wilkinson et al. (61)	RCT						✓	✓		

*GB, Gait and Balance; ULd, Upper Limb Dexterity; Bk, Bradykinesia; SV, Speech and Voice; Dy, Dysphagia; QoL, Quality of Life; Sat, Satisfaction; Feas, Feasibility; Cost*.

### Motor Symptoms

#### Gait and Balance

The search identified 2 RCTs (42, 55) and 1 case study (63) on gait and balance—Table 5. Balance and postural stability improvement after in-home VR-based balance training was the primary outcome in Gandolfi and Chatto while it was the secondary outcome in Seidler. Both RCTs compared postural stability and balance improvements of an experimental TR group with an inpatient rehabilitation group. They included 90 patients with diagnosis of PD stage 1–3 on the Hoehn and Yahr scale (H&Y) (mild to moderate PD) and *Mini-Mental State Examination (MMSE)* >24 (absence of overt dementia). The evaluation scales used in the two RCTs were: *motor sign severity (MDS-UPDRS III), Gait and balance measures (BBS; BESTest), Activities-Specific Balance Confidence (ABC), 10-Meter Walking Test (10-MWT), Dynamic Gait Index (DGI)*, and *the Parkinson's Disease Questionnaire (short version, PDQ-8)* for QoL. In detail, Gandolfi et al. (42) divided 76 PD patients (H&Y 2.5–3; MMSE ≥24/30) into two treatment groups: in-clinic SIBT (Sensory Integration Balance Program) vs. TeleWii protocol. The SIBT group (38 patients) underwent 10 balance exercises (with self-destabilization and/or external destabilization). The TeleWii group (38 patients) utilized the Nintendo Wii console for motion-controlled inputs and the Wii Fit gaming system and balance board. A laptop computer connected to a high-resolution web-camera was used to establish real-time remote visual communication via Skype software (Skype/Microsoft) between the clinic and patient's home. Ten exergames for recovery balance were developed in each session according to the patient's clinical condition and progressive improvement. Both groups performed treatment sessions of 50 min each, 3 days a week for 7 weeks.

**Table 5:** Description of the articles included focused on “Gait and Balance” as primary outcome.

	**Author**	**Pop**	**Groups**		**Inclusion Criteria**	**Training**	**Device**	**Primary Outcome**		**Secondary Outcome**		**Results**
			**C**	**Ct**				**Description**	**Scale**	**Description**	**Scale**	
Gait and balance	Gandolfi et al. (42)	76	38	38	H&Y 2.5–3; MMSE ≥ 24/30	50'/sess, 3 ds/w, 7 ws	Nintendo Wii Console + Skype	Static and dynamic balance	BBS	Balance confidence Gait speed Ability to modify gait QoL Cost analysis	ABC 10 mWT DGI PDQ-8	Improvement of static and dynamic postural control.
	Seidler et al. (55)	26	13	13	H&Y 1–3; MMSE ≥ 24/30	1 h/sess, 2 ds/w, 12 ws	Acrobat connect—PTZ pro webcam	Feasibility		Balance PD severity Gait velocity	BESTest MDS-UPDRS III GAITRite	Feasibility of TR. Improvement of balance and motor sign.
	Chatto et al. (63)	1		/		1 h/sess, 4 ds/w, 4 months	START system	Feasibility		Health-Related and Adherence Satisfaction	H&Y UPDRS PDQ-39 10 mWT TUG ABC 6 mWT Satisfaction questionnaire	Feasibility of START. Good satisfaction.

*Pop, Populations; C, Case Group; Ct, Control Group; H&Y, Hoehn & Yahr Scale; MMSE, Mini-Mental State Examination; BBS, Berg Balance Scale; ABC, Activities-Specific Balance Confidence; 10-MWT, 10-Meter Walking Test; DGI, Dynamic Gait Index; PDQ-8, Parkinson's disease Questionnaire (Short version); UPDRS, Unified Parkinson's Disease Rating Scale; START system, System for Technology-Augmented Rehabilitation and Training; PDQ-39, Parkinson's disease Questionnaire; TUG, Timed Up & Go test; 6 mWT, 6-min Walking Test*.

Seidler et al. (55) used tango dance as the rehabilitation technique. They split 26 PD patients (H&Y 1–3; MMSE ≥24/30) into two groups with non-random allocation: Telerehabilitation (Telerehab) vs. an in-person instruction group (In-person). The Telerehab group performed sessions online, connected through a private meeting room in Acrobat Connect using pro webcams. The In-person group attended instead the class sessions. With the same instructor, both groups underwent two 1-h sessions a week for 12 weeks. The main finding was that TR was feasible and produced similar improvements to face-to-face/inpatient treatment in static and dynamic postural control and balance.

The scales considered in the case study were: PD *severity (UPDRS), Activities-Specific Balance Confidence (ABC), 10-Meter Walking Test (10-MWT), Timed Up & Go (TUG) test, 6-Min Walking Test (6mWT) and the Parkinson's disease Questionnaire (PDQ-39)*.

Chatto et al. (63) presented a telehealth *System for Technology-Augmented Rehabilitation and Training* (START) to deliver the *Lee Silverman Voice Technique BIG* (LSVT BIG) therapy protocol, a physical or occupational program that improves mobility and movements used in everyday function. This system uses motion capture technologies to provide real-time and *post-hoc* feedback. A 67-year-old woman with PD at H&Y stage 2 performed 7 exercises daily organized in 16 sessions of 1 h/day, 4 times a week for 4 weeks. The main finding was that START was feasible and the patient showed high satisfaction.

#### Upper Limb Dexterity

Only one clinical trial focused on dexterity of the upper limb (UL). Cikajlo et al. (56) developed a TR exergaming system to improve UL dexterity; 26 people with PD (H&Y 2–3, MMSE <24) underwent TR treatment (an additional 2–3 weeks) after a period of inpatient training. The assessment scales used were UPDRS III, Box and Block Test (BBT), Nine-Hole Peg Test (9HPT), Jebsen's test, and PDQ-39. The results showed a short-term improvement in motor functions. In an earlier non-clinical trial, Hoffmann et al. (57) had evaluated Activities of Daily Living (ADL) (FIM, UPDRS) and hand function (9HPT) using a TR system and demonstrated that TR application can be used to produce valid and reliable assessment in people with PD—Table 6.

**Table 6:** Description of the articles included which have “Upper Limb Dexterity” as primary outcome.

	**Author**	**Pop**	**Groups**		**Inclusion Criteria**	**Training**	**Device**	**Primary Outcome**		**Secondary Outcome**		**Results**
			**C**	**Ct**				**Description**	**Scale**	**Description**	**Scale**	
UL dexterity	Cikajlo et al. (56)	28			H&Y 2–3; MMSE ≥ 24/30	30'/sess (10 EG), 7 ds/w, 3 ws	Linux operating system, Apache http server, MySQL relational database management system	Feasibility		Functional assessment	UPDRS III BBT 9HPT Jebsen's Test PDQ-39	Feasibility of TR. Improvement of motor functions and daily activity (Short-term).
	Hoffmann et al. (57)	12	6	6	Independent mobility Adequate communication and cognitive status		Low-bandwidth (18 kbit/s) public switched telephone network (PSTN) Internet connection + computer-based TR system with videoconferencing (320 × 240-pixel resolution)	ADL status	UPDRS (1-14) FIM	Hand Function [Grip strength; Pinch strength (2/3 point, lateral); Finger dexterity]	Jamar Dynamometer 9HPT	Reliability of ADL assessments and Hand function

*Pop, Populations; C, Case Group; Ct, Control Group; H&Y, Hoehn & Yahr Scale; MMSE, Mini-Mental State Examination; EG, Exergame; UPDRS, Unified Parkinson's Disease Rating Scale; BBT, Box and Block Test; 9HPT, Nine-Hole Peg Test; PDQ-39, Parkinson's disease Questionnaire; ADL, Activities of Daily Living; FIM, Functional Independence Measure*.

#### Bradykinesia

No studies dealing specifically with the treatment of bradykinesia using TR systems were found.

### Non-motor Symptoms

#### Speech and Voice

Eight articles dealing with an Internet-based TR application for the assessment of motor speech disorders in PD patients were identified—Tables 7A–C. These were 3 pilot studies [Hill et al. (65); Howell et al. (66); Quinn et al. (67)], 1 case report [Constantinescu et al. (64)], and 4 clinical trials [Constantinescu et al. (58); Constantinescu et al. (59); Theodoros et al. (60); Griffin et al. (62)]. Most of the studies used the online delivery of the Lee Silverman Voice Treatment (LSVT) LOUD program. LSVT LOUD is an intensive evidence-based speech therapy technique for motor speech and voice disorders that improves communication in daily living. This protocol requires patients to perform 16 sessions of 1 h/day, 4 times a week for 4 weeks. Research on LSVT LOUD documented that people with PD show improvements in vocal loudness and intonation of their speech, as well as speech intelligibility and voice quality (69) with short- and long-term improvements (67, 70).

**Table 7A:** Description of the articles included which have “Speech and Voice” as primary outcome.

	**Author**	**Pop**	**Groups**		**Inclusion Criteria**	**Training**	**Device**	**Primary outcome**		**Secondary outcome**		**Results**
			**C**	**Ct**				**Description**	**Scale**	**Description**	**Scale**	
Speech and voice	Constantinescu et al. (58)	61	30	31	H&Y 1–4; hypokinetic dysarthria	1 h/d, 4ds/w, 4ws + 1sess/w FtF	Personal computer-based video Conferencing system with store-and-forward capabilities, operating on a 128 kbit/s Internet connection.	Validity and reliability of online assessment	SPL ASSIDS	Satisfaction		Validity and reliability of online assessment High satisfaction.
	Constantinescu et al. (59)	34	17	17	H&Y 1–4; hypokinetic dysarthria; videolaryngoscopic evaluation	1h/d, 4ds/w, 4ws	PC-based videoconferencing (128 kbit/s Internet connection)	Validity and reliability	SPL	Satisfaction		Validity and reliability of online assessment (Non-inferiority) High satisfaction.
	Theodoros et al. (60)	51^*^	21+ 15	15	H&Y 1–5; hypokinetic dysarthria	1 h/d, 4ds/w, 4ws	eHAB (Version 2.0), a mobile multimedia TR system. Real-time videoconferencing (320 × 240 pixels), and real-time video (MPEG-4, ~768 kbit/s)	Validity (Non-Inferiority)	SPL	QoL	DIP PDQ-39	Validity of online assessment (Non-inferiority)

* *RCT Metro FTF n = 15—conventional clinic-based setting; Metro Online N = 15—treatment online; A Non-metro Online N = 21—non-randomized independent group*.

*Pop, Populations; C, Case Group; Ct, Control Group. H&Y: Hoehn & Yahr Scale; SPL, Sound Pressure Levels; ASSIDS, Assessment of Intelligibility of Dysarthic Speech; TR, Telerehabilitation; QoL, Quality of Life; DIP, Dysarthria Impact Profile; PDQ-39, Parkinson's disease Questionnaire*.

**Table 7B:** Description of the articles included which have “Speech and Voice” as primary outcome.

	**Author**	**Pop**	**Groups**		**Inclusion criteria**	**Training**	**Device**	**Primary outcome**		**Secondary outcome**		**Results**
			**C**	**Ct**				**Description**	**Scale**	**Description**	**Scale**	
Speech and voice	Griffin et al. (62)	29	8	21	Moderate hypokinetic dysarthria	LSVT program−18 sess (1 pre, 16 sess, 1 follow-up)	iPAD LSVT: “Facetime” software on Apple iPads.	Feasibility (Non-inferiority)	SPL	Cost Satisfaction		Feasibility of online assessment (non-inferiority). Improvement of SPL level. Cost reduction. High Satisfaction.
	Constantinescu et al. (64)	1		/	H&Y 1; hypokinetic dysarthria	1 h/d, 4ds/w, 4ws + 1sess/w FtF (LSVT program)	PC-based videoconferencing system (128 kb/s over public telecommunications network).	Validity and feasibility	SPL	Satisfaction		Feasibility and effectiveness of remote LSVT program. Improvement of SPL level. High satisfaction.
	Hill et al. (65)	5			Dysarthria; acquired neurological impairment	1 h/d, 4ds/w, 4ws + 1 sess/w FtF	Real-time video Conferencing at 128 kb/s	Feasibility and effectiveness	FDA (19-item) ASSIDS			Feasibility of online assessment

*Pop, Populations; C, Case Group; Ct, Control Group. LSVT, Lee Silverman Voice Treatment; SPL, Sound Pressure Levels; H&Y: Hoehn & Yahr Scale; QoL, Quality of Life; FDA, Frenchay Dysarthria Assessment*.

**Table 7C:** Description of the articles included which have “Speech and Voice” as primary outcome.

	**Author**	**Pop**	**Groups**		**Inclusion criteria**	**Training**	**Device**	**Primary outcome**		**Secondary outcome**		**Results**
			**C**	**Ct**				**Description**	**Scale**	**Description**	**Scale**	
Speech and voice	Howell et al. (66)	3		/	Hypokinetic dysarthria	1 h/d, 4ds/w, 4ws + 1sess/w FtF	1-GHz processor, 256 MB RAM, 50 MB of free disk space. Windows XP operating system with Windows Media Player, web cam	Feasibility				Feasibility of remote LSVT program (non-inferiority)
	Quinn et al. (67)	8^**^	3/3/2		H&Y 1–3; MoCA > 18; indipendent mobility, adequate communication and cognitive status	90'/sess 2 d/w 4 ws (LSVT program−8 sess)	Adobe Connect, a Logitech BC990 web camera	Feasibility (Non-Inferiority)	SPL	QoL Satisfaction	DIP PDQ-39	Feasibility of online assessment (non-inferiority). Improvement of SPL level. Improvement of QoL. High satisfaction.

** *Group one n = 3, group two n = 3, group three n = 2*.

*Pop, Populations; C, Case Group; Ct, Control Group; FtF, Face to Face. LSVT, Lee Silverman Voice Treatment; H&Y: Hoehn & Yahr Scale; MOCA, MOntreal Cognitive Assessment; SPL, Sound Pressure Levels; QoL, Quality of Life; DIP, Dysarthria Impact Profile; PDQ-39, Parkinson's disease Questionnaire*.

All pilot studies explored the feasibility and effectiveness of TR treatment for motor speech disorders. Hill et al. (65) investigated the feasibility of an Internet-based TR for treating dysarthria in 5 patients: one assessment was conducted in the traditional face-to-face manner, while the other was conducted online using the 19-item version of the Frenchay Dysarthria Assessment (FDA) and the Assessment of Intelligibility of Dysarthric Speech (ASSIDS). Howell et al. (66) delivered the LSVT program to 3 patients using Internet (broadband connection and a web camera) to treat speech disorders, measuring vocal Sound Pressure Levels (SPL) during the treatment. The result was an increase in the vocal effort and improvement in coordination. Quinn et al. (67) delivered treatment via a TR system to 8 participants who had previously received LSVT LOUD. Significant improvements were found for all SPL measures PRE-POST, which persisted in both the short- and long-term. All these pilot studies demonstrated the feasibility and the effectiveness of the online assessment of motor speech disorders. Delivering speech therapy via TR improves and maintains vocal loudness in people with PD.

Constantinescu et al. (64) explored the validity and feasibility of online delivery of LSVT in a case study using a PC-based videoconferencing system. Remote LSVT delivery proved feasible and effective. Patients reported a preference for online sessions rather than face-to-face treatment. LSVT LOUD was used to assess the participants' SPL in clinical trials. Constantinescu et al. (58) investigated the validity and reliability of a TR application for assessing speech and voice simultaneously in an online and a face-to-face environment in 61 PD patients (H&Y 1–4). The results indicated that an Internet-based assessment appears to be valid and reliable. The TR application proved to be effective to treat dysarthria. In the subsequent non-inferiority RCT, Constantinescu et al. (59) investigated the validity and reliability of online delivery of LSVT for the speech and voice disorder. 34 patients (H&Y 1–4) with hypokinetic dysarthria received LSVT in either the online or face-to-face environment. Non-inferiority of the online LSVT modality was confirmed. Online treatment for hypokinetic dysarthria associated with PD appeared to be clinically valid and reliable. Theodoros et al. (60) demonstrated non-inferiority and validity of an intensive speech treatment delivered via TR. The study was conducted on 51 PD patients (H&Y 1–5) with hypokinetic dysarthria using a mobile multimedia TR and a real-time videoconferencing system. Clinical and QoL outcomes (PDQ-39) supported the results of the intensive speech treatment. Griffin et al. (62) compared the differences in recorded speech variables between people treated with conventional “in person” LSVT to those treated remotely via iPad-based “Facetime.” Amongst 29 patients, 8 participants were selected for the iPad LSVT, while 21 joined the “in person” group. The results demonstrated a confidence interval of 90% on the measured SPL variables in both groups. Non-inferiority testing showed that the iPad LSVT is non-inferior in treating task performance measures compared to traditional LSVT.

In a grand total of 192 patients present in these 8 studies, the non-inferior effectiveness of TR was demonstrated. The online treatment of motor speech disorders with LSVT proved feasible and reliable. Improvement in the vocal pattern was a repeatable outcome of all studies, associated with a high level of patient satisfaction.

#### Dysphagia (and Swallowing)

No studies investigating functional outcomes of dysphagia were found.

### Quality of Life

Only one study by Siegert et al. (68) investigated QoL as a primary outcome—Table 8. However, QoL was investigated in other studies whose main purpose was to evaluate the efficacy of TR on motor and non-motor disorders, respectively, gait and balance (42, 63), UL dexterity (56, 57), and speech and voice (60, 67). Change in QoL was analyzed through the Parkinson's Disease Questionnaire (PDQ-39) and improvement was shown in all these studies.

**Table 8:** Description of the article included which have “Quality of Life” as primary outcome.

	**Author**	**Pop**	**Groups**		**Inclusion criteria**	**Training**	**Device**	**Primary outcome**		**Secondary outcome**		**Results**
			**C**	**Ct**				**Description**	**Scale**	**Description**	**Scale**	
Quality of life	Siegert et al. (68)		OT	UC	MoCA > 18; BBS > 41	9 months	Tablet-based + app	QoL	PDQ-8	Participation restrictions; Falling; Sleep problems; Anxiety and depression.	IMET FES-I PDSS-2 PHQ-4	(Improvement in QoL; long-term improvements and continuous care.)

*Pop, Populations; C, Case Group; Ct, Control Group; OT, Online Treatment; UC, Usual Care; MoCA, MOntreal Cognitive Assessment; PDQ-8, Parkinson's disease Questionnaire (short version); IMET, ICF-oriented instrument; FES-I, Falls Efficacy Scale International Version; PDSS-2, Parkinson's disease sleep scale; PHQ-4, Patient Health Questionnaire-4*.

Specifically, PDQ-39 is a self-report questionnaire which assesses the perception of QoL in PD patients. Consists of 39 questions investigating difficulties in 8 domains such daily activities, physical discomfort, emotional and social aspect of the disease (71).

### Patient Satisfaction

Only one study by Wilkinson et al. (61) investigated satisfaction after a telehealth program in people with PD as a primary outcome—Table 9. To assess patients satisfaction was used the Patient Assessment of Communication of Telehealth questionnaire (PACT), a 33-item validated questionnaire (Likert scale). Greater satisfaction with the telehealth modality was detected in the assessments of convenience and accessibility/distance.

**Table 9:** Description of the article included which have “Satisfaction” as primary outcome.

	**Author**	**Pop**	**Groups**		**Inclusion criteria**	**Training**	**Device**	**Primary outcome**		**Secondary outcome**		**Results**
			**C**	**Ct**				**Description**	**Scale**	**Description**	**Scale**	
Satisfaction	Wilkinson et al. (61)	86	42	42	PD; not require in-person visits; Internet access	12months	A Global Med Telehealth Specialty Carts and Cisco Webcams + Intel Health Guide	Satisfaction	PACT questionnaire	PD severity Depression QoL	UPDRS H&Y GDS PDQ-8	High satisfaction

*Pop, Populations; C, Case Group; Ct, Control Group; PD, Parkinson Disease; QoL, Quality of Life; UPDRS, Unified Parkinson's Disease Rating Scale; H&Y, Hoehn & Yahr Scale; GDS, Geriatric Depression Scale; PDQ-8, Parkinson's disease Questionnaire (short version)*.

Patient satisfaction was investigated in several studies as a secondary outcome. Research on motor (42, 63) and non-motor (58–60, 62, 64, 67) symptoms analyzed satisfaction after programs delivered by TR. In studies concerning motor aspects (gait and balance), the satisfaction was investigated using a 5-point Likert scale on a questionnaire in the RCT (42), while a semi-structured interview was conducted in the case study (63). A questionnaire with a nominal scale of 5 points was used on studies investigating non-motor aspect (speech) (58, 59, 64, 67); an opinion patients' was detected in Griffin's study (62). All studies reported high levels of agreement and patient satisfaction; only Gandolfi et al. (42) found no significant difference in satisfaction level.

### Bias Analysis

Several different biases were detected that may affect the analysis of the results. Our search identified different types of studies: 8 RCTs (42, 55–61), 1 Clinical Trial (62), 2 case studies (63, 64), 3 pilot studies (65–67), and 1 design study (68). Most trials did not blind participants and outcome assessors [UL dexterity studies (56, 57) and patient satisfaction report (61)], while the RCTs considering gait and balance (42, 55) and speech and voice (58–60) were single-blinded examiner studies. Furthermore, the analyzed reports were carried out in a variable number of participants. Of these, although some subjects affected by PD were included, the heterogeneity of the sample was noted and the inclusion criteria were variable. The range of severity of PD score measured by the Hoehn and Yahr scale was often wide: the studies on motor symptoms presented a small variability [2.5–3 (42), 1–3 (55), 2–3 (56)] while those on speech and voice dysfunction showed a larger range [1–3 (67), 1–4 (58, 59), 1–5 (60)]. The other studies do not refer to the same scale. Moreover, some studies (42, 55, 56) assessed cognitive status by MMSE, while others (67, 68) utilized the MoCA. Some of the included studies had as primary outcome the feasibility and validity of TR treatment (55–60, 62–67), while only two studies (42, 57) investigating motor symptoms in PD had as primary endpoint some clinical outcomes. Finally, motor symptoms of PD were evaluated with different scales in the different studies—details are found in Tables 5–9. In contrast, for motor speech disorders we found a greater consensus about the methods and the scales used. In summary, it was difficult to perform a robust quality analysis for each category and the dataset was considered insufficient for the planned sensitivity analyses.

## Discussion

This systematic review examined the literature to investigate whether TR leads to improvements in global or specific motor tasks (gait and balance, hand function) and non-motor dysfunction (motor speech disorders, dysphagia).

PD is a chronic degenerative pathology that leads to both motor and non-motor dysfunctions. Rehabilitation is important to improve motor function and enhance QoL. Research shows that early physical exercise is beneficial for PD patients (72). The effectiveness of sensorimotor function retraining is influenced by the quantity, duration, frequency, and intensity of exercise, and not only by the type. The feasibility and potential value of TR in people with PD has been amply demonstrated over the last decade (30, 73–77). The feasibility and accuracy of performing remote physical assessments via TR, compared with traditional face-to-face methods, showed a significantly high level of inter- and intra-rater reliability (29). Some studies evaluating the costs of telemedicine through economic analysis reported similar costs for face-to-face vs. the TR modality, but the latter saved time and cost of travel. The long-term costs have been investigated in other neurological diseases but not to date in PD dysfunction. More formal cost-benefit analysis is needed to quantify the magnitude of these benefits (29, 42, 61, 76, 78).

### Motor Symptoms

Although VR-based balance programs through TR have proved feasible and effective in several neurological conditions, in PD this system has been used separately to evaluate or to treat balance dysfunctions. Most studies related to VR application in patients with PD indicated that VR positively affected movement velocity, time, balance and gait, and postural control compared to healthy controls (79–81). Other studies that examined the effects of VR in PD patients reported a better improvement in postural stability and functioning compared to conventional treatments only (74, 82).

Others compared home-based VR training with conventional home-based balance training in patients with PD (83, 84); both groups showed similar improvements in balance and walking function. A few studies tested the Virtual Motor Rehabilitation (VMR) system, designed to improve postural control, in PD patients; preliminary results indicated improvement of balance, gait performance, and postural stability (8, 28, 81, 85, 86). To date, no study analyzed home-TR via VR systems specific for PD treatment. In fact, no specific systems have been designed. Exergames already on the market (i.e., Nintendo Wii) were used adapting them to the rehabilitation field. For example, Gandolfi et al. (42) compared improvements in postural stability after in-home VR-based balance training (TeleWii group) vs. inpatient sensory integration balance training (SIBT group). The TeleWii group performed a home-VR TR program consisting of graded exergames using the Nintendo Wii Fit system while the control group did inpatient SIBT including exercises to improve postural stability. The results suggested a similar improvement in balance (on the BBS) between the two groups.

On the contrary, studies on UL exergames found them to be acceptable and safe but they did not translate into improvement in functional activities. Tremor and rigidity in the UL can contribute to gross and fine motor coordination difficulties, which can subsequently adversely impact hand function (57). Exergames improved arm and hand activities with a home-based intervention (85). With a TR application, it was possible to perform a valid and reliable assessment of ADLs and hand function (56). Only the study by Cikajlo et al. (56) developed an intensive target-based physiotherapy for UL suitable for TR services (“FruitPicking” computer game). The TR services were comparable with usual treatment and the results showed a non-significant improvement in the dexterity of the hand.

To date only one paper (78) has evaluated the reliability and responsiveness of a motion sensor paired with a tablet app-based system for objective bradykinesia assessment both in the hospital and at home.

### Non-motor Symptoms

TR treatment studies for acquired neurologic speech disorders have mostly investigated the delivery of the LSVT LOUD. Individuals with PD are trained to “recalibrate” their motor and perceptual systems to improve self-monitoring and thus make more consistent use of the louder voice in daily communication (87). Various forms of synchronous and asynchronous technologies either as alternatives to, or in combination with, face-to-face delivery of LSVT LOUD have been investigated. The results indicate that internet-based assessment is generally reliable and valid. TR led to long-term improvements in vocal patterns and quality of voice and was well-accepted by patients with PD. The results showed high patient comfort and positive results in satisfaction. Patient perceptions have been explored (58–60, 64) using questionnaires following TR sessions. Most participants reported a positive experience and willingness to accept speech language pathology services delivered via TR (88, 89). The evidence suggests that TR will become an alternative service delivery mode for speech-language pathology.

Dysphagia is a symptom common to a wide range of medical conditions. Impairments of swallowing (dysphagia) may occur because of damage or dysfunction in the neurological control. Communication and swallowing disorders are highly prevalent in people affected by PD. Maintaining these functions over time becomes a challenge for PD patients and their caregivers. Although some emerging studies support the delivery of certain aspects of speech pathology practice via TR, the clinical assessment of dysphagia presents specific challenges for a TR model.

Only 2 preliminary reports by Sharma (90, 91) investigating patient satisfaction with regard to a specific TR-based treatment for dysphagia were identified. Sharma et al. (90) provided pilot information on the basic feasibility and validity of conducting dysphagia assessments via TR and examined the potential of TR for swallowing disorders. Ten simulated patients were assessed simultaneously face-to-face and by TR. The results were positive, with high levels of agreement observed between both groups on all parameters of interest (oromotor function and swallowing). Sharma et al. (91) evaluated patients' perception pre-treatment and their satisfaction after the TR treatment through pre- and post-session questionnaires in 40 patients with dysphagia (4 with PD). The 14 questions explored comfort with the use of TR, satisfaction, benefits of TR assessments, and the patient-preferred assessment modality. The results demonstrated that the use of TR in speech-language pathology, specifically in the assessment, and management of swallowing disorders, is a promising approach. It however requires insight from patients and clinicians in order to achieve optimal care. Patients had positive changes in their pre-assessment perceptions and had high levels of satisfaction with their experience. The data were positive and highlighted that patients are interested in and willing to receive services via TR. This provided preliminary evidence for the feasibility of remote dysphagia assessment. In the literature, only preliminary data collections in patients following laryngectomy were identified by Ward et al. (89). Recent evidence supports the feasibility, validity, and reliability of administering clinical dysphagia assessments via TR as opposed to face-to-face in several neurological conditions including PD (90–92).

### QoL and Patient Satisfaction

PD can significantly alter the capacity to perform regular ADLs (e.g., self-care tasks) as well as work and leisure activities. Reduced independence in ADLs has been linked to a poorer QoL in people with PD (57). Some studies have evaluated how TR programs and, more generally, telemedicine can influence the QoL. Siegert et al. (68) investigated QoL as a primary outcome and found that providing remote specialist assistance directly into people's homes is feasible and improves healthcare, QoL, and social participation. These results show that a tablet-based training program can help to maintain long-term functional ability for PD. Dorsey et al. (75), in a telemedicine study, evaluated if the remote delivery of specialty care directly into people's homes can enhance access and improve the healthcare of individuals with chronic conditions, via virtual house calls. The authors showed that telemedicine can improve participants' QoL. QoL was also investigated in studies whose aim was to study the influence of TR on motor (30, 42, 56, 63) and non-motor symptoms (60, 67). The results indicate a short-term improvement in the QoL.

In addition, a few studies on motor (42, 63) and non-motor (58, 59, 64) symptoms have investigated patients' satisfaction with a TR program, reporting positive results. Although these results are positive, the number of studies analyzing patient satisfaction in association with TR is still too small to draw any meaningful conclusions. However, several studies exist that reported patient satisfaction following a telemedicine program. The study of Wilkinson et al. (61) is a dual-arm RCT focused on patient satisfaction as the primary endpoint (Patient Assessment of Communication of Telehealth questionnaire—PACT), as well as on clinical outcomes, patient travel burden, and health care utilization, using clinical video telehealth vs. usual inpatient care. Telemedicine studies, such as Venkataraman et al. (93), sought to characterize the recommendations and feedback of patients with PD obtained following a free consultation with a specialist via a virtual visit. The studies found high patient satisfaction with telehealth. Two studies by Sharma et al. (90) and Sharma et al. (91) had as primary endpoint the satisfaction of patients following participation in assessment of dysphagia conducted by TR. The studies showed patients' comfort and high satisfaction. Feasibility studies reported patient satisfaction following a telemedicine intervention.

High patient satisfaction was found in the studies by Antonini et al. (76) and Ferreira et al. (77), which also highlighted the ease of use of the treatment system. Barbour et al. (29) analyzed satisfaction not only of the patients, but also of family members, sub-specialists and nursing staff, reporting the same results.

### Limitations and Strengths of TR in PD

TR systems for people with PD show some limitations. First, feasibility of TR is good in PD early stages and adult patients (not too old): patient's age, stage of disease and cognitive status are fundamental factors determining the success of a TR program. The majority of the studies cited in the present review found use of TR for assessment and treatment to be valid and reliable in the early stages of PD, but reported that it may not apply to individuals at an advanced stage of PD. To date, the findings regarding TR are similar to those for face-to-face treatment but there are no studies evaluating the long-term effectiveness of TR systems on motor symptoms. Secondly, long-term follow up are not available: while studies have demonstrated TR systems to be effective in the management of musculoskeletal disorders and neurological pathologies, in PD only preliminary studies are available, carried out on small numbers of patients. Finally, motor symptoms of PD (balance, gait, postural instability, UL dexterity) were evaluated with different scales in the different studies and different types of devices were applied (often not specifically set for PD patients), making it difficult to analyze accurately across the board the effectiveness of the TR systems. In contrast, for motor speech disorders a greater consensus about the methods, the scales and the devices used was evident.

The positive aspects of these preliminary studies are that at first TR has been proven to be suitable to treat both motor and non-motor symptoms in PD patients. Secondly, the majority of the studies reported here considered and measured the improvement in QoL and patient satisfaction after treatment.

### Future Researce

Regarding future studies, based on the evidence to date, we would make two main recommendations. The standardization of the assessment scales used for PD motor symptoms is highly recommended. The scales most commonly used are: PDQ for QoL, UPDRS (embraces motor symptoms but does not embrace the pathology in all its complexity); BEST/mini-BESTest, TUG, 10-MWT, ABC for gait and balance and postural instability; 9HPT for UL dexterity. On the contrary, studies on non-motor symptoms (speech and voice) used a standardized system for research, which led to results that are more meaningful. Further studies on the efficacy of TR in the management of motor and non-motor symptoms of PD are also necessary.

## Conclusion

Telerehabilitation is a solution for delivering services at home, supporting patients and clinicians by minimizing the barriers of distance, time, and cost. TR applications may increase the accuracy of assessment scoring and therefore provide more precise information for diagnostic and treatment purposes. Home-based assessment, compared to patients reporting to a clinic, allows more frequent evaluations, greater consistency, and adherence to therapy changes. The availability of low-cost home-based solutions for the reliable and automated assessment of motor and non-motor symptoms in PD is highly desirable due to the advantages it offers. This analysis highlighted that TR systems with VR and wearable sensors are effective in maintaining and/or improving some clinical aspects of PD (gait and balance, speech and voice, QoL, patient satisfaction). The TR model opens up new opportunities for treating PD patients enabling the delivery of rehabilitation care with a reduction of patient discomfort, although no proven superiority of a TR treatment over face-to-face has been demonstrated yet.

### Key Message

This systematic review suggests that TR in PD patients is indicated in the early stages of disease and in particular in adult patients with preserved cognitive status.

As a general outcome, the indication is to carry out the TR treatment for 1 h/day, 3–4 days/weeks, for 4–12 weeks.

TR was demonstrated to be efficient in the improvement of specific outcomes since feedback or augmented reality embedded in this particular rehab-technique help the training of specific gestures stimulated by the achievement of a target, the correct execution of the movement and self-management.

This review highlighted that in order to improve the effectiveness of the treatment, a key factor is the use of devices that incorporate advanced technologies like visual/auditory feedback and augmented reality in order to specifically train a task. However, a comparative study amongst different technologies does not exist yet.

For the clinician it is of paramount importance to dispose of standardized and effective assessment tools (scales) and devices that use the same technology in order to normalize the treatment on a set of patients.

The patient must have an easy-to-use tool that provides feedback to guide him toward the achievement of the goal.

Through augmented feedback (VR, biofeedback, etc.) used in technologically assisted rehabilitation it was possible to whiteness an increase of the patient compliance and to train successfully tasks that are generally unaware like daily and sport gestures.

## Data Availability Statement

The raw data supporting the conclusions of this article will be made available by the authors, without undue reservation.

## Author Contributions

CV, GM, and SB contributed to conception and design of the study. CV and SB organized the database. AG and RC checked data. CV wrote the first draft of the manuscript. GM, SB, and FB wrote sections of the manuscript. All authors contributed to manuscript revision, read, and approved the submitted version.

## Conflict of Interest

The authors declare that the research was conducted in the absence of any commercial or financial relationships that could be construed as a potential conflict of interest.

## Publisher's Note

All claims expressed in this article are solely those of the authors and do not necessarily represent those of their affiliated organizations, or those of the publisher, the editors and the reviewers. Any product that may be evaluated in this article, or claim that may be made by its manufacturer, is not guaranteed or endorsed by the publisher.

## Abbreviations

PD: Parkinson Disease
QoL: Quality of Life
ICT: Information and Communication Technologies
TR: Telerehabilitation
VR: Virtual Reality
RCT: Randomized Controlled Trial
ADL: Activities of Daily Living
H&Y: Hoehn & Yahr Scale
MMSE: Mini-Mental State Examination
BBS: Berg Balance Scale
10-MWT: 10-Meter Walking Test
DGI: Dynamic Gait Index
TUG: Timed Up & Go test
6mWT: 6-min Walking Test
MDS-UPDRS III: Unified Parkinson's Disease Rating Scale
ABC: Activities-Specific Balance Confidence
PDQ: Parkinson's disease Questionnaire
UL: Upper Limbs
BBT: Box and Block Test
9HPT: Nine-Hole Peg Test
FIM: Functional Independence Measure
LSVT: Lee Silverman Voice Treatment
SPL: Sound Pressure Levels.

## Appendix 1

Search strategy: take the search process via PUBMED as an example.

“Parkinson's Disease” OR “Parkinson Disease” OR PD

AND

Telerehabilitation OR “Remote Rehabilitation” OR “home-based rehabilitation” OR telehealth OR telemedicine

Filters Used:

- Article Type: Clinical Trial, Randomized Controlled Trial, Review, Systematic Review, Meta-Analysis
- Publication Date: studies released between January 2005 and December 2019
- Language: English
- Age: Adults 19+

## References

[1] De Lau LM Breteler MM. Epidemiology of Parkinson's disease. Lancet Neurol. (2006) 5:525–35. 10.1016/S1474-4422(06)70471-9 16713924

[2] Balestrino R Schapira AHV. Parkinson disease. Europ J Neurol. (2020) 27:27–42. 10.1111/ene.14108 31631455

[3] Nussbaum RL Ellis CE. Alzheimer's disease and Parkinson's disease. N Engl J Med. (2003) 348:1356–64. 10.1056/NEJM2003ra020003 12672864

[4] Lee A Gilbert RM. Epidemiology of Parkinson disease. Neurol Clin. (2016) 34:955–65. 10.1016/j.ncl.2016.06.012 27720003

[5] Pringsheim T Jette N Frolkis A Steeves TD. The prevalence of Parkinson's disease: a systematic review and meta-analysis. Move Disord. (2014) 29:1583–90. 10.1002/mds.25945 24976103

[6] von Campenhausen S Bornschein B Wick R Bötzel K Sampaio C Poewe W. Prevalence and incidence of Parkinson's disease in Europe. Europ Neuropsychopharmacol. (2005) 15:473–90. 10.1016/j.euroneuro.2005.04.007 15963700

[7] Gillies GE Pienaar IS Vohra S Qamhawi Z. Sex differences in Parkinson's disease. Front Neuroendocrinol. (2014) 35:370–84. 10.1016/j.yfrne.2014.02.002 24607323 PMC4096384

[8] Albiol Pérez S Gil-Gómez JA Gil-Gómez H Muñoz-Tomás MT Vial-Escolano R Lozano-Quilis JA. The Effect of balance training on postural control in patients with Parkinson's disease using a virtual rehabilitation system. Methods Inf Med. (2017) 56:138–44. 10.3414/ME16-02-0004 28244545

[9] Fahn S. Description of Parkinson's disease as a clinical syndrome. Ann N Y Acad Sci. (2003) 991:1–4. 10.1111/j.1749-6632.2003.tb07458.x 12846969

[10] Chaudhuri KR Odin P Antonini A Martinez-Martin P. Parkinson's disease: the non-motor issues. Parkinsonism Relat Disord. (2011) 17:717–23. 10.1016/j.parkreldis.2011.02.018 21741874

[11] Schapira AH Chaudhuri KR Jenner P. Non-motor features of Parkinson disease. Nat Rev Neurosci. (2017) 18:435. 10.1038/nrn.2017.62 28592904

[12] Pinto S Cardoso R Sadat J Guimarães I Mercier C Santos H. Dysarthria in individuals with Parkinson's disease: a protocol for a binational, cross-sectional, case-controlled study in French and European Portuguese (FraLusoPark). BMJ Open. (2016) 6:e012885. 10.1136/bmjopen-2016-012885 27856480 PMC5128890

[13] Matsubara T Suzuki K Fujita H Watanabe Y Sakuramoto H Matsubara M. Autonomic symptoms correlate with non-autonomic non-motor symptoms and sleep problems in patients with Parkinson's disease. Europ Neurol. (2018) 80:193–9. 10.1159/000495797 30572329

[14] Salat-Foix D Suchowersky O. The management of gastrointestinal symptoms in Parkinson's disease. Expert Rev Neurotherap. (2012) 12:239–48. 10.1586/ern.11.192 22288679

[15] Blackett H Walker R Wood B. Urinary dysfunction in Parkinson's disease: a review. Parkinsonism Relat Disord. (2009) 15:81–7. 10.1016/j.parkreldis.2007.10.016 18474447

[16] Goldstein DS Holmes C Li ST Bruce S Metman LV Cannon RO III. Cardiac sympathetic denervation in Parkinson disease. Ann Internal Med. (2000) 133:338–47. 10.7326/0003-4819-133-5-200009050-00009 10979878

[17] Ondo WG Vuong KD Khan H Atassi F Kwak C Jankovic J. Daytime sleepiness and other sleep disorders in Parkinson's disease. Neurology. (2001) 57:1392–6. 10.1212/WNL.57.8.1392 11673578

[18] Nolano M Provitera V Estraneo A Selim MM Caporaso G Stancanelli A. Sensory deficit in Parkinson's disease: evidence of a cutaneous denervation. Brain. (2008) 131:1903–11. 10.1093/brain/awn102 18515869

[19] Lin CH Lin JW Liu YC Chang CH Wu RM. Risk of Parkinson's disease following anxiety disorders: a nationwide population-based cohort study. Europ J Neurol. (2015) 22:1280–7. 10.1111/ene.12740 26031920

[20] Marin RS Fogel BS Hawkins J Duffy J Krupp B. Apathy: a treatable syndrome. J Neuropsychiat Clin Neurosci. (1995) 7:23–30. 10.1176/jnp.7.1.23 7711487

[21] Weintraub D Moberg PJ Duda JE Katz IR Stern MB. Effect of psychiatric and other nonmotor symptoms on disability in Parkinson's disease. J Am Geriatrics Soc. (2004) 52:784–8. 10.1111/j.1532-5415.2004.52219.x 15086662

[22] Nombela C Bustillo PJ Castell P Medina V Herrero MT. Cognitive rehabilitation in Parkinson's disease: evidence from neuroimaging. Front Neurol. (2011) 2:82. 10.3389/fneur.2011.00082 22203816 PMC3244758

[23] Jankovic J Aguilar LG. Current approaches to the treatment of Parkinson's disease. Neuropsychiatric Dis Treat. (2008) 4:743. 10.2147/NDT.S2006 19043519 PMC2536542

[24] Magrinelli F Picelli A Tocco P Federico A Roncari L Smania N. Pathophysiology of motor dysfunction in Parkinson's disease as the rationale for drug treatment and rehabilitation. Parkinson's Dis. (2016) 2016:9832839. 10.1155/2016/9832839 27366343 PMC4913065

[25] Geroin C Gandolfi M Bruno V Smania N Tinazzi M. Integrated approach for pain management in Parkinson disease. Curr Neurol Neurosci Rep. (2016) 16:28. 10.1007/s11910-016-0628-7 26879763

[26] Oguh O Eisenstein A Kwasny M Simuni T. Back to the basics: regular exercise matters in Parkinson's disease: results from the National Parkinson Foundation QII registry study. Parkinsonism Related Disord. (2014) 20:1221–5. 10.1016/j.parkreldis.2014.09.008 25258329

[27] Grazina R Massano J. Physical exercise and Parkinson's disease: influence on symptoms, disease course and prevention. Rev Neurosci. (2013) 24:139–52. 10.1515/revneuro-2012-0087 23492553

[28] Pachoulakis I Xilourgos N Papadopoulos N Analyti A. A Kinect-based physiotherapy and assessment platform for Parkinson's disease patients. J Med Engin. (2016) 2016:2795090. 10.1155/2016/9413642 27822467 PMC5086395

[29] Barbour PJ Arroyo J High S Fichera LB Staska-Pier MM McMahon MK. Telehealth for patients with Parkinson's disease: delivering efficient and sustainable long-term care. Hosp Pract. (2016) 44:92–7. 10.1080/21548331.2016.1166922 26982525

[30] Bphty MN Boccthy LT Boccthy AV. Internet-based physical assessment of people with Parkinson disease is accurate and reliable: a pilot study. J Rehabil Res Dev. (2013) 50:643. 10.1682/JRRD.2012.08.0148 24013912

[31] Intzandt B Beck EN Silveira CR. The effects of exercise on cognition and gait in Parkinson's disease: a scoping review. Neurosci Biobehav Rev. (2018) 95:136–69. 10.1016/j.neubiorev.2018.09.018 30291852

[32] Linares-del Rey M Vela-Desojo L Cano-de la Cuerda R. Mobile phone applications in Parkinson's disease: a systematic review. Neurologí*a*. (2019) 34:38–54. 10.1016/j.nrleng.2018.12.002 28549757

[33] Ben-Pazi H Browne P Chan P Cubo E Guttman M Hassan A. The promise of telemedicine for movement disorders: an interdisciplinary approach. Curr Neurol Neurosci Rep. (2018) 18:26. 10.1007/s11910-018-0834-6 29654523

[34] Achey MA Beck CA Beran DB Boyd CM Schmidt PN Willis AW. Virtual house calls for Parkinson disease (Connect. Parkinson): study protocol for a randomized, controlled trial. Trials. (2014) 15:1–3. 10.1186/1745-6215-15-465 25431346 PMC4289172

[35] Gregory P Alexander J Satinsky J. Clinical telerehabilitation: applications for physiatrists. PM&R. (2011) 3:647–56. 10.1016/j.pmrj.2011.02.024 21777864

[36] Agostini M Moja L Banzi R Pistotti V Tonin P Venneri A. Telerehabilitation and recovery of motor function: a systematic review and meta-analysis. J Telemed Telecare. (2015) 21:202–13. 10.1177/1357633X15572201 25712109

[37] McCue M Fairman A Pramuka M. Enhancing quality of life through telerehabilitation. Phys Med Rehabil Clin. (2010) 21:195–205. 10.1016/j.pmr.2009.07.005 19951786

[38] Hailey D Roine R Ohinmaa A Dennett L. Evidence of benefit from telerehabilitation in routine care: a systematic review. J Telemed Telecare. (2011) 17:281–7. 10.1258/jtt.2011.101208 21844172

[39] Galea MD. Telemedicine in rehabilitation. Phys Med Rehabil Clin. (2019) 30:473–83. 10.1016/j.pmr.2018.12.002 30954160

[40] Peretti A Amenta F Tayebati SK Nittari G Mahdi SS. Telerehabilitation: review of the state-of-the-art and areas of application. JMIR Rehabil Assistive Technol. (2017) 4:e7. 10.2196/rehab.7511 28733271 PMC5544892

[41] Horak FB. Postural orientation and equilibrium: what do we need to know about neural control of balance to prevent falls?. Age Ageing. (2006) 35(suppl_2):ii7–11. 10.1093/ageing/afl077 16926210

[42] Gandolfi M Geroin C Dimitrova E Boldrini P Waldner A Bonadiman S. Virtual reality telerehabilitation for postural instability in Parkinson's disease: a multicenter, single-blind, randomized, controlled trial. BioMed Res Int. (2017) 2017:7962826. 10.1155/2017/7962826 29333454 PMC5733154

[43] Piron L Turolla A Tonin P Piccione F Lain L Dam M. Satisfaction with care in post-stroke patients undergoing a telerehabilitation programme at home. J Telemed Telecare. (2008) 14:257–60. 10.1258/jtt.2008.080304 18633001

[44] Giansanti D Tiberi Y Silvestri G Maccioni G. Toward the integration of novel wearable step-counters in gait telerehabilitation after stroke. Telemed e-Health. (2009) 15:105–11. 10.1089/tmj.2008.0051 19199855

[45] Hermens H Huijgen B Giacomozzi C Ilsbroukx S Macellari V Prats E. Clinical assessment of the HELLODOC tele-rehabilitation service. Ann Ist Super Sanita. (2008) 44:154–63. 18660565

[46] Amatya B Galea MP Kesselring J Khan F. Effectiveness of telerehabilitation interventions in persons with multiple sclerosis: a systematic review. Multiple Sclerosis Relat Disord. (2015) 4:358–69. 10.1016/j.msard.2015.06.011 26195057

[47] Robb JF Hyland MH Goodman AD. Comparison of telemedicine versus in-person visits for persons with multiple sclerosis: a randomized crossover study of feasibility, cost, and satisfaction. Multiple Sclerosis Relat Disord. (2019) 36:101258. 10.1016/j.msard.2019.05.001 31472419

[48] Piotrowicz E Piotrowicz R. Cardiac telerehabilitation: current situation and future challenges. Europ J Prevent Cardiol. (2013) 20(2_suppl):12–16. 10.1177/2047487313487483c 23702985

[49] Tousignant M Boissy P Moffet H Corriveau H Cabana F Marquis F. Patients' satisfaction of healthcare services and perception with in-home telerehabilitation and physiotherapists' satisfaction toward technology for post-knee arthroplasty: an embedded study in a randomized trial. Telemed e-Health. (2011) 17:376–82. 10.1089/tmj.2010.0198 21492030

[50] Palacín-Marín F Esteban-Moreno B Olea N Herrera-Viedma E Arroyo-Morales M. Agreement between telerehabilitation and face-to-face clinical outcome assessments for low back pain in primary care. Spine. (2013) 38:947–52. 10.1097/BRS.0b013e318281a36c 23238489

[51] Pani D Piga M Barabino G Crabolu M Uras S Mathieu A. Home tele-rehabilitation for rheumatic patients: impact and satisfaction of care analysis. J Telemed Telecare. (2017) 23:292–300. 10.1177/1357633X16632950 26945913

[52] Finkelstein J Lapshin O Castro H Cha E Provance PG. Home-based physical telerehabilitation in patients with multiple sclerosis: a pilot study. J Rehabil Res Dev. (2008) 45:1361–73. 10.1682/JRRD.2008.01.0001 19319760

[53] Marshall SG Shaw DK Honles GL Sparks KE. Interdisciplinary approach to the rehabilitation of an 18-year-old patient with bronchopulmonary dysplasia, using telerehabilitation technology. Respirat Care. (2008) 53:346–50. 18291051

[54] Moher D Liberati A Tetzlaff J Altman DG Prisma Group. Preferred reporting items for systematic reviews and meta-analyses: the PRISMA statement. PLoS Med. (2009) 6:e1000097. 10.1371/journal.pmed.1000097 19621072 PMC2707599

[55] Seidler KJ Duncan RP McNeely ME Hackney ME Earhart GM. Feasibility and preliminary efficacy of a telerehabilitation approach to group adapted tango instruction for people with Parkinson disease. J Telemed Telecare. (2017) 23:740–6. 10.1177/1357633X16668092 27624469

[56] Cikajlo I Hukić A Dolinšek I Zajc D Vesel M Krizmanič T. Can telerehabilitation games lead to functional improvement of upper extremities in individuals with Parkinson's disease?. Int J Rehabil Res. (2018) 41:230. 10.1097/MRR.0000000000000291 29757774 PMC6092088

[57] Hoffmann T Russell T Thompson L Vincent A Nelson M. Using the internet to assess activities of daily living and hand function in people with Parkinson's disease. NeuroRehabilitation. (2008) 23:253–61. 10.3233/NRE-2008-23307 18560142

[58] Constantinescu G Theodoros D Russell T Ward E Wilson S Wootton R. Assessing disordered speech and voice in Parkinson's disease: a telerehabilitation application. Int J Lang Commun Disord. (2010) 45:630–44. 10.3109/13682820903470569 20102257

[59] Constantinescu G Theodoros D Russell T Ward E Wilson S Wootton R. Treating disordered speech and voice in Parkinson's disease online: a randomized controlled non-inferiority trial. Int J Lang Commun Disord. (2011) 46:1–16. 10.3109/13682822.2010.484848 21281410

[60] Theodoros DG Hill AJ Russell TG. Clinical and quality of life outcomes of speech treatment for Parkinson's disease delivered to the home via telerehabilitation: a noninferiority randomized controlled trial. Am J Speech-Lang Pathol. (2016) 25:214–32. 10.1044/2015_AJSLP-15-0005 27145396

[61] Wilkinson JR Spindler M Wood SM Marcus SC Weintraub D Morley JF. High patient satisfaction with telehealth in Parkinson disease: a randomized controlled study. Neurology. (2016) 6:241–51. 10.1212/CPJ.0000000000000252 27347441 PMC4909521

[62] Griffin M Bentley J Shanks J Wood C. The effectiveness of Lee Silverman Voice Treatment therapy issued interactively through an iPad device: a non-inferiority study. J Telemed Telecare. (2018) 24:209–15. 10.1177/1357633X17691865 28147896

[63] Chatto CA York PT Slade CP Hasson SM. Use of a telehealth system to enhance a home exercise program for a person with Parkinson disease: a case report. J Neurol Phys Ther. (2018) 42:22–9. 10.1097/NPT.0000000000000209 29206708

[64] Constantinescu GA Theodoros DG Russell TG Ward EC Wilson SJ Wootton R. Home-based speech treatment for Parkinson's disease delivered remotely: a case report. J Telemed Telecare. (2010) 16:100–4. 10.1258/jtt.2009.090306 20008051

[65] Hill AJ Theodoros DG Russell TG Cahill LM Ward EC Clark KM. An Internet-based telerehabilitation system for the assessment of motor speech disorders: a pilot study. Am J Speech Lang Pathol. (2006) 15:45–56. 10.1044/1058-0360(2006/006) 16533092

[66] Howell S Tripoliti E Pring T. Delivering the Lee Silverman Voice Treatment (LSVT) by web camera: a feasibility study. Int J Lang Commun Disord. (2009) 44:287–300. 10.1080/13682820802033968 18821113

[67] Quinn R Park S Theodoros D Hill AJ. Delivering group speech maintenance therapy via telerehabilitation to people with Parkinson's disease: a pilot study. Int J Speech-Lang Pathol. (2019) 21:385–94. 10.1080/17549507.2018.1476918 29879854

[68] Siegert C Hauptmann B Jochems N Schrader A Deck R. ParkProTrain: an individualized, tablet-based physiotherapy training programme aimed at improving quality of life and participation restrictions in PD patients–a study protocol for a quasi-randomized, longitudinal and sequential multi-method study. BMC Neurol. (2019) 19:1–9. 10.1186/s12883-019-1355-x 31238908 PMC6593548

[69] Ramig L Halpern A Spielman J Fox C Freeman K. Speech treatment in Parkinson's disease: randomized controlled trial (RCT). Move Disord. (2018) 33:1777–91. 10.1002/mds.27460 30264896 PMC6261685

[70] Ramig LO Countryman S O'Brien C Hoehn M Thompson L. Intensive speech treatment for patients with Parkinson's disease: short-and long-term comparison of two techniques. Neurology. (1996) 47:1496–504. 10.1212/WNL.47.6.1496 8960734

[71] Jenkinson C Fitzpatrick RAY Peto VIV Greenhall R Hyman N. The Parkinson's Disease Questionnaire (PDQ-39): development and validation of a Parkinson's disease summary index score. Age Ageing. (1997) 26:353–7. 10.1093/ageing/26.5.353 9351479

[72] Cai G Huang Y Luo S Lin Z Dai H Ye Q. Continuous quantitative monitoring of physical activity in Parkinson's disease patients by using wearable devices: a case-control study. Neurol Sci. (2017) 38:1657–63. 10.1007/s10072-017-3050-2 28660562

[73] Dorsey ER Deuel LM Voss TS Finnigan K George BP Eason S. Increasing access to specialty care: a pilot, randomized controlled trial of telemedicine for Parkinson's disease. Move Disord. (2010) 25:1652–9. 10.1002/mds.23145 20533449

[74] Dorsey ER Venkataraman V Grana MJ Bull MT George BP Boyd CM. Randomized controlled clinical trial of “virtual house calls” for Parkinson disease. JAMA Neurol. (2013) 70:565–70. 10.1001/jamaneurol.2013.123 23479138 PMC3791511

[75] Dorsey ER Achey MA Beck CA Beran DB Biglan KM Boyd CM. National randomized controlled trial of virtual house calls for people with Parkinson's disease: interest and barriers. Telemed e-Health. (2016) 22:590–8. 10.1089/tmj.2015.0191 26886406 PMC4939367

[76] Antonini A Gentile G Giglio M Marcante A Gage H Touray MM. Acceptability to patients, carers and clinicians of an mHealth platform for the management of Parkinson's disease (PD_Manager): study protocol for a pilot randomised controlled trial. Trials. (2018) 19:1–11. 10.1186/s13063-018-2767-4 30217235 PMC6138904

[77] Ferreira JJ Godinho C Santos AT Domingos J Abreu D Lobo R. Quantitative home-based assessment of Parkinson's symptoms: the SENSE-PARK feasibility and usability study. BMC Neurol. (2015) 15:1–7. 10.1186/s12883-015-0343-z 26059091 PMC4460963

[78] Cubo E Mariscal N Solano B Becerra V Armesto D Calvo S. Prospective study on cost-effectiveness of home-based motor assessment in Parkinson's disease. J Telemed Telecare. (2017) 23:328–338. 10.1177/1357633X16638971 27000142

[79] Santos Mendes FAD Pompeu JE Lobo AM Silva KGD Oliveira TDP Zomignani AP. Motor learning, retention and transfer after virtual-reality-based training in Parkinson's disease-effect of motor and cognitive demands of games: a longitudinal, controlled clinical study. Physiotherapy. (2012) 98:217–23. 10.1016/j.physio.2012.06.001 22898578

[80] Arroyo-Gallego T Ledesma-Carbayo MJ Sánchez-Ferro A Butterworth I Mendoza CS Matarazzo M. Detection of motor impairment in Parkinson's disease via mobile touchscreen typing. IEEE Trans Biomed Eng. (2017) 64:1994–2002. 10.1109/TBME.2017.2664802 28237917

[81] Ferraris C Nerino R Chimienti A Pettiti G Cau N Cimolin V. Feasibility of home-based automated assessment of postural instability and lower limb impairments in Parkinson's disease. Sensors. (2019) 19:1129. 10.3390/s19051129 30841656 PMC6427119

[82] Lee NY Lee DK Song HS. Effect of virtual reality dance exercise on the balance, activities of daily living, and depressive disorder status of Parkinson's disease patients. J Phys Ther Sci. (2015) 27:145–147. 10.1589/jpts.27.145 25642060 PMC4305547

[83] Yang WC Wang HK Wu RM Lo CS Lin KH. Home-based virtual reality balance training and conventional balance training in Parkinson's disease: a randomized controlled trial. J Formosan Med Assoc. (2016) 115:734–43. 10.1016/j.jfma.2015.07.012 26279172

[84] Song J Paul SS Caetano MJD Smith S Dibble LE Love R. Home-based step training using videogame technology in people with Parkinson's disease: a single-blinded randomised controlled trial. Clin Rehabil. (2018) 32:299–311. 10.1177/0269215517721593 28745063

[85] Fung A Lai EC Lee. A new smart balance rehabilitation system technology platform: development and preliminary assessment of the Smarter Balance System for home-based balance rehabilitation for individuals with Parkinson's disease. In: 2018 40th Annual International Conference of the IEEE Engineering in Medicine and Biology Society (EMBC). Honolulu: IEEE (2018). p. 1534–7. 10.1109/EMBC.2018.8512528 30440685

[86] Allen NE Song J Paul SS Smith S O'Duffy J Schmidt M. An interactive videogame for arm and hand exercise in people with Parkinson's disease: a randomized controlled trial. Parkinsonism Relat Disord. (2017) 41:66–72. 10.1016/j.parkreldis.2017.05.011 28528804

[87] Cherney LR Halper AS Kaye RC. Computer-based script training for aphasia: emerging themes from post-treatment interviews. J Commun Disord. (2011) 44:493–501. 10.1016/j.jcomdis.2011.04.002 21612787

[88] Brennan DM Georgeadis AC Baron CR Barker LM. The effect of videoconference-based telerehabilitation on story retelling performance by brain-injured subjects and its implications for remote speech-language therapy. Telemed J e-Health. (2004) 10:147–54. 10.1089/tmj.2004.10.147 15319044

[89] Ward E Crombie J Trickey M Hill A Theodoros D Russell T. Assessment of communication and swallowing post-laryngectomy: a telerehabilitation trial. J Telemed Telecare. (2009) 15:232–7. 10.1258/jtt.2009.081204 19590028

[90] Sharma S Ward EC Burns C Theodoros D Russell T. Assessing swallowing disorders online: a pilot telerehabilitation study. Telemed e-Health. (2011) 17:688–95. 10.1089/tmj.2011.0034 21882996

[91] Sharma S Ward EC Burns C Theodoros D Russell T. Training the allied health assistant for the telerehabilitation assessment of dysphagia. J Telemed Telecare. (2012) 18:287–91. 10.1258/jtt.2012.111202 22790011

[92] Theodoros D Aldridge D Hill AJ Russell T. Technology-enabled management of communication and swallowing disorders in Parkinson's disease: a systematic scoping review. Int J Lang Commun Disord. (2019) 54:170–88. 10.1111/1460-6984.12400 29923267

[93] Venkataraman V Donohue SJ Biglan KM Wicks P Dorsey ER. Virtual visits for Parkinson disease: A case series. Neurol Clin Pract. (2014) 4:146–52. 10.1212/01.CPJ.0000437937.63347.5a 24790799 PMC4001180

